# Anais Brasileiros de Dermatologia. Beginning a new five-year term, 2026-2030

**DOI:** 10.1016/j.abd.2025.501287

**Published:** 2026-01-23

**Authors:** Hiram Larangeira de Almeida Jr.

**Affiliations:** Universidade Católica de Pelotas, Pelotas, RS, Brazil

In 2026, a new five-year editorial period begins in the *Anais Brasileiros de Dermatologia.*

The group that starts now consists of myself as Scientific Editor with three Associate Editors: Jane Tomimori from Universidade Federal de São Paulo, Neusa Yuriko Sakai Valente from Universidade de São Paulo, and Renata Ferreira Magalhães from Universidade Estadual de Campinas.

Our mission will be to continue the work of the previous group,^1^, ^2^ valuing the quality of the selected articles, contributing solidly to the dissemination of scientific progress.

In the text excerpt depicted below (Fig. 1), from the preface of the fourth edition of the *Précis de Dermatologie* of 1928, Darier expresses his concern, almost a century ago, to fill the knowledge gaps,^3^ which we do to this day, in every research and in every publication.

**Figure 1:**
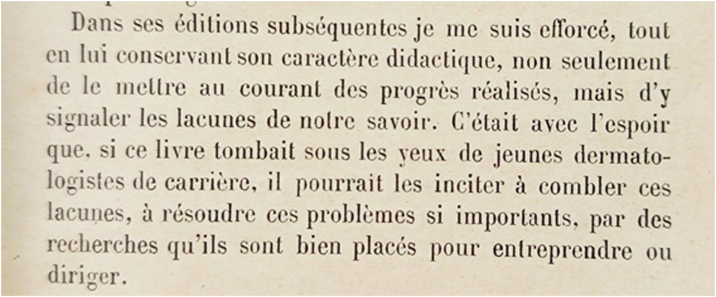
Excerpt from the preface to the fourth edition of *Précis de Dermatologie of 1928.*

Each piece of information that we make available to the global dermatological community helps to fill gaps and complete the complex understanding of skin diseases, which is experiencing so many diagnostic and therapeutic advances.

“In subsequent editions, while maintaining its didactic characteristics, I have strived not only to make known the progress made, but also to point out the gaps in our knowledge. This is done in the hope that if this book falls before the eyes of young dermatologists, it may encourage them to fill these gaps, to solve these truly important problems, through research that they undertake.” This should be the legend of Fig. 1

## Research data availability

Not applicable.

## Financial support

None declared.

## Author’ contributions

Hiram Larangeira de Almeida Junior: Approval of the final version of the manuscript; drafting and editing of the manuscript.

## Conflicts of interest

None declared.
